# Paint it Black: Using Change-Point Analysis to Investigate Increasing Vulnerability to Depression towards the End of Vincent van Gogh’s Life

**DOI:** 10.3390/healthcare5030053

**Published:** 2017-09-04

**Authors:** Arnold A. P. van Emmerik, Ellen L. Hamaker

**Affiliations:** 1 Department of Clinical Psychology, Faculty of Social and Behavioural Sciences, University of Amsterdam, Nieuwe Achtergracht 129, Amsterdam 1018 WS, The Netherlands; 2 Department of Methodology and Statistics, Faculty and Social of Behavioural Sciences, Utrecht University, Padualaan 14, Utrecht 3584 CH, The Netherlands; e.l.hamaker@uu.nl

**Keywords:** depression, self-focus, change-point analysis, time series, arts, Vincent van Gogh

## Abstract

This study investigated whether Vincent van Gogh became increasingly self-focused—and thus vulnerable to depression—towards the end of his life, through a quantitative analysis of his written pronoun use over time. A change-point analysis was conducted on the time series formed by the pronoun use in Van Gogh’s letters. We used time as a predictor to see whether there was evidence for increased self-focus towards the end of Van Gogh’s life, and we compared this to the pattern in the letters written before his move to Arles. Specifically, we examined Van Gogh’s use of first person singular pronouns (FPSP) and first person plural pronouns (FPPP) in the 415 letters he wrote while working as an artist before his move to Arles, and in the next 248 letters he wrote after his move to Arles until his death in Auvers-sur-Oise. During the latter period, Van Gogh’s use of FPSP showed an annual increase of 0.68% (*SE* = 0.15, *p* < 0.001) and his use of FPPP showed an annual decrease of 0.23% (*SE* = 0.04, *p* < 0.001), indicating increasing self-focus and vulnerability to depression. This trend differed from Van Gogh’s pronoun use in the former period (which showed no significant trend in FPSP, and an annual increase of FPPP of 0.03%, *SE* = 0.02, *p* = 0.04). This study suggests that Van Gogh’s death was preceded by a gradually increasing self-focus and vulnerability to depression. It also illustrates how existing methods (i.e., quantitative linguistic analysis and change-point analysis) can be combined to study specific research questions in innovative ways.

## 1. Introduction

Vincent van Gogh’s life, his work, and the circumstances surrounding his death continue to interest a large audience today. One of the most persisting questions concerns the nature of Van Gogh’s physical and mental symptoms. Various diagnoses have been put forward [1,2,3], none of which (with the exception of bipolar disorder) preclude a comorbid diagnosis of major depressive disorder. Despite its obvious appeal, the “depression hypothesis” has received little attention in the scientific literature. This may be largely explained by the limited diagnostic information that is available, including a few 19th century medical records, the symptom descriptions in Van Gogh’s letters, and observations from his relatives and acquaintances [4]. The current study proposes a unique and innovative approach to investigate the “depression hypothesis” through statistically analyzing certain linguistic characteristics of Van Gogh’s letters.

Depression has been theoretically linked to elevated self-focused attention [5], which in turn may be linguistically operationalized by an increased use of first person singular pronouns (FPSP; i.e., I, me, mine), and a decreased use of first person plural pronouns (FPPP; i.e., we, us, our) [6]. Several empirical studies have provided evidence for a relationship of FPSP and FPPP with depression, as well as suicide [7,8,9]. To investigate the hypothesis that Van Gogh—in addition to suffering from possible other disorders—became increasingly vulnerable to depression towards the end of his life, we therefore studied his use of these pronouns.

Specifically, we focused on Van Gogh’s pronoun use in the letters he wrote between his move to Arles on 21 February 1888, and his death almost two and a half years later, on 29 July 1890, in Auvers-sur-Oise. Van Gogh’s move to Arles has been recognized as a crucial change in his artistic and personal life. Initially, his arrival in Arles was characterized by strong optimism, as evidenced by for instance his plans to establish an artist community. However, it also marked the beginning of a series of adversities that may have functioned as triggers for depression, including Van Gogh’s fall out with fellow artist Paul Gauguin, and the onset of his ominous ‘attacks’ and repeated hospitalizations. We therefore hypothesized that the letters that Van Gogh wrote after his move to Arles are characterized by an increase in FPSP and a decrease in FPPP, reflecting a gradual increase in self-focused attention. Moreover, we expected this trend to differ from Van Gogh’s pronoun use in the letters he wrote before his move to Arles (between June 1880, when he took up painting, and February 1888).

## 2. Materials and Methods 

Van Gogh’s letters were accessed through http://www.vangoghletters.org/vg/ [10] and analyzed with the Linguistic Inquiry and Word Count (LIWC2007) program [11]. LIWC2007 computes the proportions to which a text file contains words from more than 60 word categories, including FPSP and FPPP. Dutch, French, and English dictionaries are available in LIWC2007, thus allowing for the analysis of Van Gogh’s letters in their original languages. A total of 668 letters were written by Van Gogh between June 1880 and July 1890. Five of these letters contained less than 25 words, and were excluded because of the disproportionately large weights of individual words in these texts. Of the 663 remaining letters, 415 letters were written before Van Gogh’s move to Arles and 248 were written afterwards; 509 letters were addressed to his brother Theo and 154 to other persons; and 419 letters were written in Dutch, 239 in French, and 5 in English. 

We modeled the percentage of FPSP and FPPP in each letter using change-point analysis (also referred to as structural break analysis, piecewise or segmented regression, or turning point analysis [12]). This approach allowed for separate underlying trends in Van Gogh’s pronoun use before and after his move to Arles, so that we could determine whether there were changes in these trends as well as sudden changes associated with the move. Because pronoun use may partly depend on addressee (i.e., letters to his brother Theo may be more intimate and include more FPSP and FPPP than letters to other persons), and may inherently differ between languages, we controlled for addressee and language to rule out possible confounding effects of these variables on Van Gogh’s pronoun use.

## 3. Results

**Figure 1:**
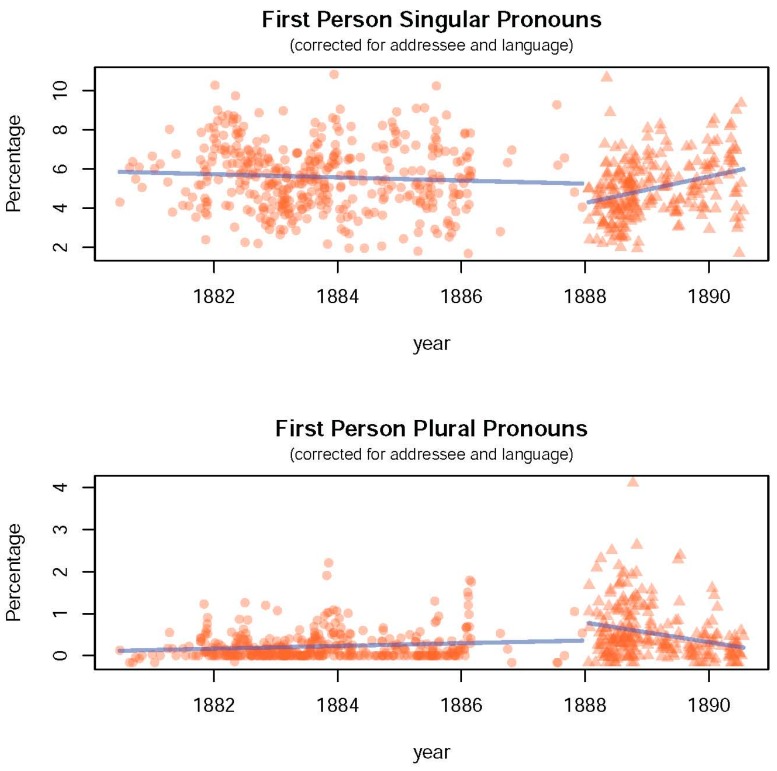
Percentage of first person singular pronouns and first person plural pronouns per letter (corrected for addressee and language). Blue lines represent trends before (dots) and after (triangles) Van Gogh’s move to Arles.

The results of the change-point analyses are presented in Table 1. These show that after Van Gogh’s move to Arles, his letters are characterized by an *annual increase* of 0.68% for FPSP (*SE* = 0.15, *p* < 0.001), and an *annual decrease* of 0.23% for FPPP (*SE* = 0.04, *p* < 0.001), which is consistent with a pattern of increasing self-focused attention. These slopes differ significantly from the slopes before the move, as indicated by the estimated *change in slopes* between the second and the first phase (i.e., a positive change of 0.76%, *SE* = 0.15, *p* < 0.001 for FPSP, and a negative change of −0.27%, *SE* = 0.05, *p* < 0.001 for FPPP). Furthermore, the move itself is associated with a *sudden decrease* in FPSP (−0.95%, *SE* = 0.42, *p* = 0.022), and a *sudden increase* in FPPP (0.42%, *SE* = 0.12, *p* = 0.001), which may reflect Van Gogh’s initial optimism surrounding the move. Figure 1 contains the percentage of FPSP and FPPP in each letter (corrected for addressee and language) plotted against time, and shows the underlying trends.

There are no significant differences in FPSP and FPPP between Van Gogh’s letters to Theo and to others. Language only affects FPSP, which was 1.49% higher in English letters than in Dutch letters (*SE* = 0.72, *p* = 0.04). Finally, the adjusted R^2^ for FPSP is only 0.04 (i.e., a small effect size), while for FPPP it is 0.23 (i.e., a medium to large effect size). In conclusion, the results support the hypothesis that Van Gogh became more self-focused, and thereby more vulnerable to depression, during his stay in Arles. Furthermore, the move to Arles constituted a significant change-point in Van Gogh’s life with respect to his self-focused attention.

## 4. Discussion

We found the changes in Van Gogh’s pronoun use towards the end of his life to be consistent with the self-regulatory perseveration theory of depression [13], which assigns a central role to self-focused attention in the development and maintenance of depression. According to this theory, self-focused attention magnifies the negative affect that occurs after the loss of a valued source of identity or self-esteem (in Van Gogh’s case, for example, brought about by the adversities that he encountered upon moving to Arles, as described above), ultimately resulting in the negative self-image that characterizes depression. With regard to word use, this model predicts that depressed individuals’ language is characterized by higher levels of FPSP and by lower levels of FPPP.

To our knowledge, the present methodology has not been applied to Van Gogh’s letters before, and direct comparisons with previous quantitative studies of his word use are therefore not possible. However, our findings converge with a growing body of empirical research on the associations of pronoun use to depression and suicide. Closely paralleling our findings, an idiographic study of the diary of Italian writer Cesare Pavese, for instance, found a gradual increase of FPSP in the year that preceded his suicide [14]. Furthermore, suicide notes contained more personal pronouns than legacy tokens of spree killers or student writings [15]. Furthermore, reductions of depressive symptoms were associated with reductions of the use of “I” over time in an expressive writing treatment for female survivors of childhood sexual abuse [16]. Finally, a recent meta-analysis confirmed the presence of an association between FPSP and depressive symptoms, which was robust across a number of moderators including age, gender, type of sample (clinical versus non-clinical), language mode (written versus spoken), and setting (private versus interpersonal language) [17].

The current study’s major strength is its hitherto unique combination of computerized word-use analysis and change-point analysis to explore unresolved research questions in new ways (in this case, Van Gogh’s mental health status at the end of his life). While people consciously control the words they use to communicate their thoughts and feelings to others, nevertheless, some aspects of word use, including the use of pronouns, are likely to escape conscious control. This study shows how this notion, which dates back to the origins of clinical psychology [18], can be made the subject of modern-day technological and statistical methods. An important limitation is that the utility and validity of this particular combination of techniques now needs further corroboration in live individuals, including the use of previously validated assessment methods to evaluate its diagnostic potential.

## 5. Conclusions

There is a growing body of empirical research that supports the theory of a positive link between elevated self-focused attention, which manifests itself through a particular use of first person pronouns, and vulnerability to depression and suicide. In the current study we found that Van Gogh’s use of FPSP and FPPP in his letters reflects an increasing self-focus in the final years of his life leading up to his suicide attempt. Moreover, this gradual increase was not present before our a priori hypothesized change point (i.e., his move to Arles and the ensuing adversities). In sum, while our analyses cannot prove that Van Gogh was clinically depressed at the time of his death, we conclude that his first person pronoun use is in line with a gradually increasing self-focus and thus vulnerability to depression and suicide towards the end of his life

## Figures and Tables

**Table 1:** Results from change-point analyses of the percentage of first person singular and plural pronouns (FPSP and FPPP, respectively) in Van Gogh’s letters. Slopes represent annual change in percentage of FPSP and FPPP use.

		Slope phase 1	Slope phase 2	Change in slope	Sudden change	Theo vs. other	French vs. Dutch	English vs. Dutch
**FPSP**	Estimate (SE) *p*-value	−0.08 (0.06) 0.14	0.68 (0.15) < 0.001	0.76 (0.15) < 0.001	−0.95 (0.42) 0.02	0.27 (0.15) 0.08	0.56 (0.30) 0.06	1.49 (0.72) 0.04
**FPPP**	Estimate (SE) *p*-value	0.03 (0.02) 0.04	−0.23 (0.04) < 0.001	−0.27 (0.05) < 0.001	0.41 (0.12) < 0.01	-0.00 (0.05) 0.97	0.16 (0.09) 0.06	0.14 (0.21) 0.49
